# Supplementary material for: Predicting visual function by interpreting a neuronal wiring diagram
Source: Nature. 2024 Oct 2;634(8032):113–23. doi: 10.1038/s41586-024-07953-5 (PMC11446822; doi:10.1038/s41586-024-07953-5)
Supplement: Supplementary file 6 — CRF and ERF predictions for individual TmY4 and TmY9 cells. Analogous to Supplementary Data 3, but for TmY target types. Shown are the top four monosynaptic pathways, the strongest pathway passing through each of the top ten intermediary types (ranking from Extended Data Fig. 7), and the trisynaptic pathway Tm1–TmY–Dm3–TmY (see the section entitled Prediction of spatial normalization). [file 41586_2024_7953_MOESM6_ESM.zip › DataS4/TmY9q/720575940624187573.pdf]

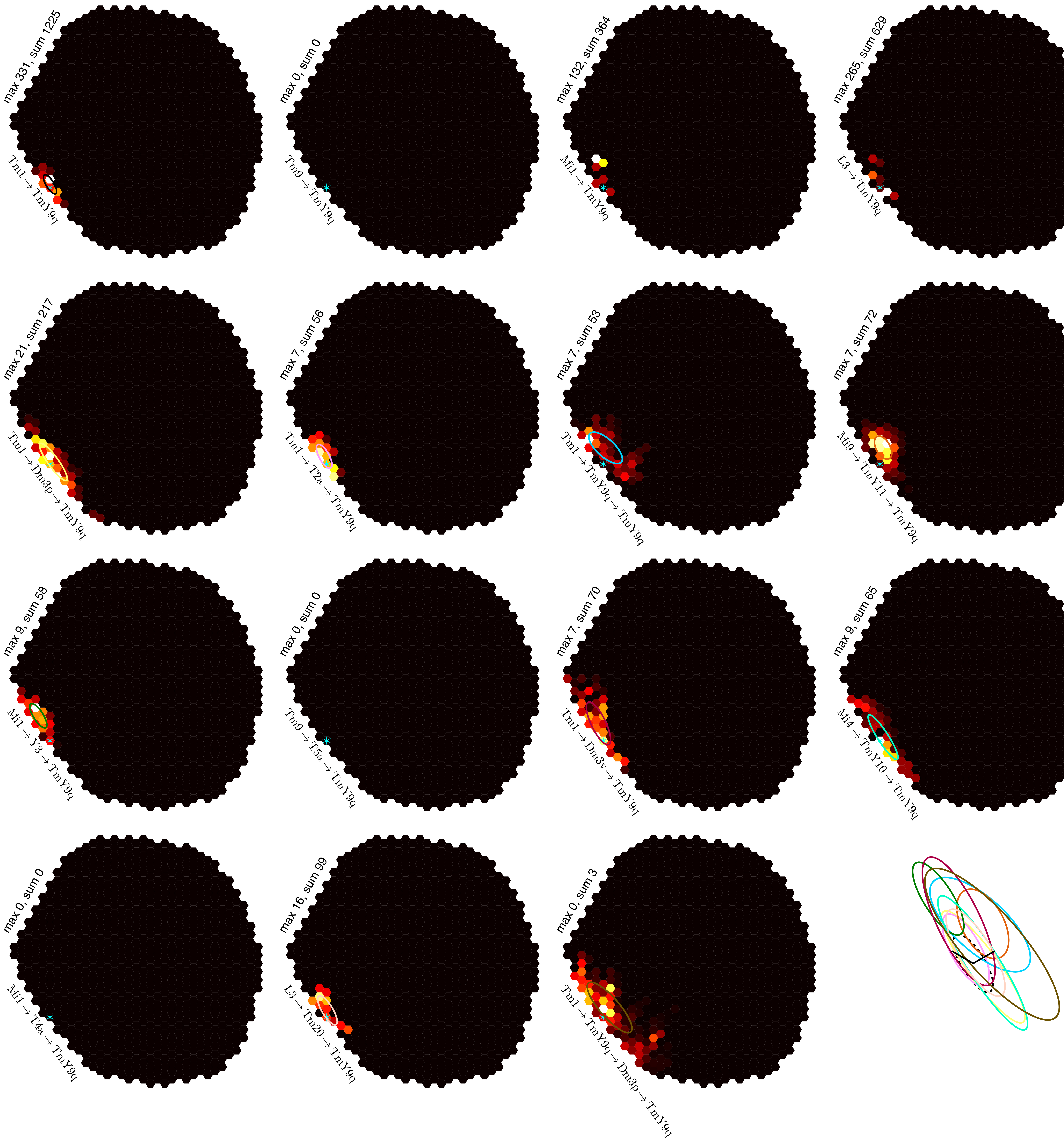
max 331, sum 1225
Tm1 → TmY9q
max 0, sum 0
Tm9 → TmY9q
max 132, sum 364
Mi1 → TmY9q
max 265, sum 629
L3 → TmY9q
max 21, sum 217
Tm1 → Dm3p → TmY9q
max 7, sum 56
Tm1 → T2a → TmY9q
max 7, sum 53
Tm1 → TmY9q → TmY9q
max 7, sum 72
Mi9 → TmY11 → TmY9q
max 9, sum 58
Mi1 → Y3 → TmY9q
max 0, sum 0
Tm9 → T5a → TmY9q
max 7, sum 70
Tm1 → Dm3v → TmY9q
max 9, sum 65
Mi4 → TmY10 → TmY9q
max 0, sum 0
Mi1 → T4a → TmY9q
max 16, sum 99
L3 → Tm20 → TmY9q
max 0, sum 3
Tm1 → TmY9q → Dm3p → TmY9q
